# Hydrogel-Based Novel Biomaterials: Achievements and Prospects

**DOI:** 10.3390/gels10070436

**Published:** 2024-06-29

**Authors:** Ana Paula Serro, Diana Cristina Silva, Ana Isabel Fernandes

**Affiliations:** 1 Centro de Química Estrutural (CQE), Institute of Molecular Sciences, Instituto Superior Técnico, Universidade de Lisboa, 1049-001 Lisboa, Portugal; 2 Egas Moniz Center for Interdisciplinary Research (CiiEM), Egas Moniz School of Health & Science, Campus Universitário, 2829-511 Caparica, Portugal

In recent decades, hydrogels have garnered significant attention, thanks to their extensive biomedical and pharmaceutical applications [1,2]. These remarkable materials closely mimic biological tissues and exhibit unique behaviors due to their high-water content. The ability to customize their properties and enhance cell interactions by selecting specific monomers and synthesis techniques has propelled hydrogels to the forefront of biomaterial innovation.

Hydrogels’ versatility has led to successful applications in areas as diverse as injectable particulate systems, contact lenses, cartilage substitutes, catheter linings, valves, suture threads, wound-healing dressings, skin grafts, and biosensors [3]. Furthermore, hydrogels are playing an increasingly vital role in tissue engineering, regenerative medicine, and targeted drug delivery systems, cementing their status as a cornerstone of modern biomedical science [4]. Figure 1 illustrates some of the areas where hydrogels are most applicable. 

Although significant efforts have been made to develop new hydrogels with improved properties and additional functionalities, several challenges remain and are currently the focus of intense research. These are being addressed through a multidisciplinary approach, combining knowledge of biology, chemistry, materials engineering and other disciplines, such as pharmaceutics. One major hurdle to overcome is the biocompatibility of hydrogels. Ensuring that hydrogels do not provoke adverse immune responses and can integrate seamlessly with biological tissues is crucial for their effective use in medical applications. Additionally, the mechanical properties of hydrogels need to be finely tuned to match the specific requirements of the different administration routes/tissues and clinical applications. For example, hydrogels used in cartilage replacement must be strong and elastic, while those used in wound dressings should be flexible and breathable. Depending on the case, other features may be of major importance, e.g., oxygen permeability, transparency or tribological behavior. Hydrogels with cross-linking/gelation capacity, which allows their formation in situ at the target site, may be advantageous in some situations. Appropriate degradation rates and bioactive surfaces that promote vascularization and support suitable tissue architecture may also be desirable. In all cases, the ability to prevent colonization by microorganisms, thereby reducing the risk of infection, is an added value. Their resistance to sterilization methods is also a transversal requirement, to ensure that they remain functional and safe after the sterilization procedures. Finaly, researchers are exploring new synthesis and functionalization methods, as well as novel materials to make hydrogel manufacturing more efficient and affordable [5]. The scalability and cost-effectiveness of hydrogel production are essential for their widespread clinical adoption.

This Special Issue presents a selection of the cutting-edge research that is being carried out by teams from diverse countries across Europe, Asia and America with the objective of developing new hydrogels that can meet the aforementioned requirements. It gathers eight original research articles covering topics that range from the production of hydrogels, addressing for example the influence of the nature of the raw materials and of the incorporation of additives, to the evaluation of the effects of unconventional sterilization methods. The characterization of the materials and their behavior is achieved through in vitro testing, experiments with animals and clinical trials. The presented works cover not only biotechnological applications, such as the immobilization of enzymes, scaffolds for cellular metabolism studies or tissue engineering, and biofabrication, but also therapeutic applications, e.g., ocular, local anti-cancer treatments and topical or transdermal drug delivery (Figure 2). The latter application is the most representative and covers areas such as wound or burn healing and treatment of sports injuries. The two reviews presented at the end of this collection provide an overview of the latest advances in the production and therapeutic potential of hydrogels in wound healing and cancer treatment. A summary of each of the works published in the Special Issue is given below. 

The first article evaluates the effect of the origin and variability of the raw materials on the synthesis of gelatin methacryloyl (GelMA) and production of hydrogels. Eight types of gelatin, from distinct sources (five porcine, one from fish, and two bovine) and with different bloom values, were made to react with methacrylic anhydride in the presence of urea to maintain room temperature and avoid GelMA gelation. The degree of functionalization (DoF) of the obtained product was influenced by the bloom strength and by the gelatin’s donor species. Furthermore, the characteristics of the product were also affected by the batch-to-batch variability of the gelatins. In the second stage of the work, the authors produced hydrogel discs by UV photopolymerization using the synthesized GelMAs and investigated their viscoelastic behavior, concluding that it depends on the protein’s DoF, bloom strength and origin. Additionally, a microfluidic device was used to generate droplets of GelMA solutions that were subsequently crosslinked to obtain hydrogel microparticles. The droplet size was controlled through the ratio continuous phase (GelMA solution) to the dispersed phase (sunflower seed oil). The swelling capacity of the obtained microparticles varied inversely with GelMA concentration. As a potential use of the microparticles, the authors advocate for cellular expansion and differentiation in stirred bioreactors, due to the high surface to volume ratio and to the fact that GelMA presents cell adhesion sites. Comparatively to underivatized gelatin, the proposed microparticles are easier to produce, as the crosslinking can be performed in a single stage by photopolymerization.

In another work, thermoresponsive hydrogels based on Pluronic^®^ F127 containing different polysaccharides (xanthan gum, alginate, κ-carrageenan, gellan, or chitosan) were prepared for possible application in injectable biomaterials or bioinks. The rheological behavior of the materials was studied in different shear conditions and aqueous environments. Pluronic^®^ F127 formed micellar networks with self-healing capacity, since after being submitted to high strain cycles, they were able to recover the initial structure. The viscoelastic behavior of the hydrogels containing xanthan gum was found to depend on the testing liquid. They exhibited shear-thinning behavior, yield stress, and enhanced self-healing capabilities. The experiments revealed that the best results were obtained for a xanthan gum concentration of 1%. However, the addition of salt led to aggregate formation and diminished the hydrogel performance. Hydrogels containing the same content of the remaining natural polymers were also tested. Although good results have been obtained with the hydrogels containing alginate, κ-carrageenan and gellan gum, their ability to recover the initial structure after application of high strains decreased. The addition of chitosan implied the use of acetic acid for its solubilization, which must have been responsible for the inferior viscoelastic performance and self-healing ability shown. The authors concluded that the hydrogel’s structure and respective rheological properties can be tuned by choosing the appropriate polysaccharide. 

Covering different aspects of the hydrogel production process, this Special Issue also includes an article on sterilization. This is a mandatory step to obtain biologically safe materials that can be implanted/used in direct contact with the body. The sterilization method and conditions must not only fulfill the sterility requirement but also ensure that the material maintains adequate properties for the intended application. Many hydrogels are thermo- and/or radiosensitive, precluding the use of standard industrial methods and warranting the quest for alternative sterilization methods. Pires et al. investigated the effect of three non-conventional methods, (a) microwave (MW); (b) high hydrostatic pressure (HHP), and (c) plasma (PM), on the properties of reinforced polyvinyl alcohol (PVA) hydrogels intended for articular cartilage replacement. The material was reinforced with poly(p-phenylene-2,6-benzobisoxazole) (PBO) nanofibers to assure the high mechanical performance needed in this demanding application. All the methods were effective at achieving the materials’ sterility and did not affect the hydrogels’ water content, nor their hydrophilicity. However, they induced some changes in crystallinity and/or crosslinking. MW was revealed to be the most suitable method for the studied hydrogel, since it further improved its mechanical properties, namely the hardness, stiffness and shear modulus. In addition, it reduced the friction coefficient observed against natural cartilage. The sterilized hydrogel kept its non-irritant and non-cytotoxic behavior. MW is deemed a suitable method for the sterilization of this type of hydrogel, due to the ease of use, low cost and short processing time (3 min). 

Illustrating the application of hydrogels with bioactive agents for therapeutic purposes, Francisco et al. used Pluronic^®^ F127 to produce a hydrogel that, due to the incorporation of silver nanoparticles (AgNPs), may be used in skin burn regeneration. In fact, AgNPs have demonstrated antibacterial activity and are promising in wound healing, a major public health issue lacking effective therapeutic strategies. In this work, different methods of nanoparticle production were tested and the one that yielded less aggregates and more homogeneous particles (dispersion index < 0.2) was selected. The AgNPs produced were characterized by atomic force microscopy (AFM), diffraction laser scattering (DLS) and zeta potential measurements. AgNPs were non-spherical, with an average size of 48.04 ± 14.87 nm and slightly negative surface charge (−0.79 ± 2.17); their solution was translucent yellow, with an absorption peak at 407 nm. Pluronic^®^ F127, an amphiphilic poly(ethylene oxide)/poly(propylene oxide)/poly(ethylene oxide) triblock copolymer, was used to produce a thermoreversible hydrogel (i.e., upon administration, at body temperature, a stable gel matrix is formed), incorporating the AgNPs. Antibacterial activity was shown in vitro against known colonizers of infected skin burns (*Escherichia coli*, *Staphylococcus aureus* and *Pseudomonas aeruginosa*). In vitro skin permeation studies, using an artificial membrane to mimic the skin, demonstrated no AgNP permeation after 24 h, a good safety indicator. The semi-solid formulation was tested in vivo (mice) in a chemical skin burn model and its therapeutic effect and safety were compared to a commercial silver sulfadiazine cream. The skin regeneration performance of the topical hydrogel-loaded AgNPs at lower silver doses was comparable to the more concentrated commercial formulation, demonstrating the clinical potential of this approach.

Traditional hydrogels, despite their ability to absorb large amounts of water, typically exhibit delayed swelling after application and are not suitable for the treatment of moderate to heavily exuding wounds. Pinthong and co-workers developed a porous hydrogel incorporating Manuka honey (MH, 1 and 10% *w*/*w*) for such use. The hydrogel matrix was based on 2-acrylamido-2-methyl-1-propanesulphonic acid with the addition of pore-forming excipients—sodium bicarbonate (foaming agent), methacrylic acid (to promote CO_2_ formation during gelation), and Poloxamer 407 (foam stabilizer)—to create a structure capable of rapidly absorbing significant amounts of water. The formation of a gas-blown porous hydrogel, with pores ranging from ~50 to 110 µm (scanning electron microscopy; SEM), the superior bulk swelling performance (~5000% vs. ~2000% increase in weight, by gravimetry) and initial surface absorption (10 μL vs. <1 μL, in <3000 ms, by contact angle measurement) compared to the non-porous counterpart were confirmed. Incorporation of MH resulted in smaller (~50 μm) and more homogeneous pores, increased porosity, more linear swelling behavior in the first 5 min, improved gel appearance and mechanical properties. The inherent properties of MH, which altered the system by increasing viscosity and lowering the pH, enhancing foam production during gelation, appear to be responsible for the improvements observed. Cell cytotoxicity was tested by the XTT (2,3-bis-(2-methoxy-4-nitro-5-sulfophenyl)-2H-tetrazolium-5-carboxanilide) assay, using normal human dermal fibroblasts, to evaluate cell viability during 24 h. Despite the acidic nature and hydrogen peroxide and flavonoid content of MH, cell viability was ~80-90% and the materials were regarded as non-toxic. In conclusion, the porous hydrogels fabricated offer a promising approach for advanced rapid-absorbing wound dressings.

To minimize discomfort, swelling, and inflammation following sports or accident injuries, non-steroidal anti-inflammatory drugs are typically administered. Permeation of the drug into the damaged tissues is paramount and dependent on formulation. The following work by Bukhari et al. evaluated the efficacy of diclofenac potassium (DK 2, 4 and 6%) gels, with and without phonophoresis application, in the management of such injuries. Marketed gels of the sodium salt of the drug (DS, 4%) and drug-free gels were used as controls. The patients (*n* = 200) were randomly allocated into five groups (*n* = 20 each) to which the different formulations were administered 3–4 times weekly, for 4 weeks, without phonophoresis; for another five similar groups, ultrasounds were applied in continuous mode (2:1), at a frequency of 0.8 MHz and an intensity of about 1.5 W/cm^2^, to maximize the effect of the topical formulation. The patients were assessed (at baseline and sessions 4, 8, 12 and 16) by using the Numeric Pain Rating Scale (NPRS) and the Western Ontario McMaster Osteo-Arthritis (WOMAC) indexes for pain in disability and stiffness. Both scales showed significant dose-dependent pain relief in DK-treated groups, as compared to the group treated with DS gel. The additional application of phonophoresis resulted in faster and deeper pain relief due to the increased penetration of both DK and DS gels; the significant increases in benefits were dose-dependent and particularly stronger for DK gels. Moreover, phonophoresis was well tolerated by patients. The results demonstrated the superiority of the therapeutic scheme combining 6% DK gel and a physical enhancer of drug permeation, which is particularly suitable for the treatment of uncomplicated soft tissue injuries (e.g., plantar fasciitis, bursitis stress injuries, and tendinitis). 

Glaucoma is a neurodegenerative disorder that may lead to vision loss or blindness because of damage to the optic nerve. One of the most significant risk factors for developing the disease is elevated intraocular pressure, which may be prevented using anti-hypertensive beta-adrenergic blockers such as carvedilol. However, due to its poor water solubility and extensive first-pass metabolism, carvedilol demonstrates low oral bioavailability, necessitating the use of alternative drug delivery routes (e.g., topical ocular) and formulations capable of remaining at the target site for long periods of time. To this end, Almutairy et al. fabricated a thermosensitive in situ gelling system for the ophthalmic delivery of carvedilol-loaded spanlastics (CRV-SPLs). SPLs are elastic colloidal carriers based on non-ionic surfactants and an edge activator, with enhanced corneal permeability of the entrapped drugs. The incorporation of such vesicles into an in situ forming hydrogel is anticipated to facilitate prolonged drug release, accurate dosage, straightforward administration, prolonged residence time and enhanced transcorneal permeation. Optimized SPLs with minimal diameter, high entrapment efficiency and drug permeability were prepared with Span 60:Brij 97 (80:20). Gels were formulated with thermosensitive polymers (poloxamer 407/poloxamer 188) and processing parameters (e.g., gelation temperature, muco-adhesion, rheological properties, and in vitro drug release pattern) were optimized. The gels produced were transparent (slightly less after loading with SPLs) with acceptable pH for ocular use (~neutrality) and high drug content (>97.3%). Ex vivo drug permeation studies using goat corneal membrane showed a marked drug permeation increase due to the nanoencapsulation in SPLs. Visual appearance, drug content, pH, and gelling capability were maintained over a period of 8 weeks, at 4 °C. In vivo pharmacokinetics, based on calculations of the amount of CRV quantified in the aqueous humor of rabbits over time, after a single administration, was analyzed using a non-compartment model. A rapid onset of action, and increased drug residence time and penetration through cornea were observed, resulting in higher AUC, t_1/2_ and C_max_. The optimized carvedilol-loaded in situ gel system was found to be efficacious in lowering intra ocular pressure, for up to 8 h after instillation, demonstrating its sustained action. Irritation, evaluated by the Draize rabbit eye test, was ruled out. In conclusion, the results demonstrated the potential of CRV-loaded spanlastic vesicles to enhance ocular bioavailability and serve as an alternative to conventional dosage forms in glaucoma treatment.

The high area/mass ratio and reactivity of polymeric nanofibers have been explored in controlled release and targeting of biomolecules in several diseases including cancer. Guerra and co-workers fabricated electrospun polyvinyl alcohol (PVA) and PVA/chitosan (CS) hydrogel nanofiber systems of lysozyme (Lys), crosslinked with boronic acids and functionalized with magnetic iron oxide nanoparticles (IONPs), for biological applications. The antibiotic, antitumor and immunomodulatory properties of Lys justify the choice of this enzyme. Magnetic nanovesicles have found usefulness in hyperthermia-based cancer therapy due to the sensitivity of some cancer cells to temperatures > 41 °C. Application of an alternating magnetic field to IONPs results in a temperature increase and irreversible damage and selective death of tumor cells (damage to normal cells is reversible). Electrospinning solutions were characterized in terms of specific conductivity and surface tension; morphological analysis of the electrospun nanofibers, as a function of processing parameters, was conducted by SEM. Most of the nanofibers were white, uniform, relatively thin and fragile and circular in shape (diameter ~ 3.3 cm) with an average mass of 4.12 mg. The joint use of PVA and CS seemed to improve the chemical and resistance properties of the nanofibers; crosslinking with boronic acids reduced its fragility. The effects of temperature and pH on the in vitro release and biological activity of the immobilized enzyme were determined by the *Micrococcus lysodeikticus* method. The nanofibers showed controlled release and Lys exhibited a high release rate at pH 6.74 and 45.5 °C. Since cancer cells exhibit lower pH and are more sensitive to temperature than normal cells, these features may prove useful in anticancer applications. The presence of IONPs does not seem to influence Lys release, but crosslinking is essential for controlled release over time. Enzymatic activity, ascertained by microbial lysis, presented a lag time and was not affected by the presence of magnetic particles. The cytotoxicity of the systems was evaluated in a widely used model of the intestinal mucosal barrier, an epithelial Caco-2 cell line, from colon carcinoma. After 10 days of contact, cell viability was evaluated by the MTT (3-[4,5-dimethylthiazol-2-yl]-2,5 diphenyl tetrazolium bromide) assay and these systems were found to be cytotoxic, especially those coated with IONPs. The biodegradability and bioresponsiveness of the nanofibers at high temperature highlight their potential for in situ application to reduce or inhibit the process of tumor metastasis.

Moving to the literature reviews, Marques et al. collected information on the recent advances over the past two decades, regarding the development of injectable poloxamer hydrogels for anti-cancer local treatments. Local cancer therapies offer several advantages relatively to systemic treatments, e.g., intravenous administration. In fact, intra/peri-tumoral injections ensure high levels of the therapeutic agents at the target site, while avoiding systemic toxicity, and their effect does not depend on the tumor vasculature. Injectable biodegradable hydrogels present the added advantage of eliminating the need for surgery and increase the retention of the drug in the tumor. Poloxamer-based gelling systems have raised special interest due to their biocompatibility, ease of preparation, thermoresponsiveness, and ability to incorporate various anticancer agents. The review describes the physicochemical and biological properties of injectable poloxamer hydrogels and summarizes their applications in local cancer therapy, namely in chemotherapy, phototherapy, immunotherapy, and gene therapy. Despite the promising preclinical results, such treatments are still at the proof-of-concept stage. To advance towards their use, poloxamers must be modified/combined with other polymers to reduce erosion and ensure precise drug delivery. Additionally, a more extensive characterization of the hydrogels is needed. Besides evaluating the rheological and biological behavior, insights into morphology and thermal properties should also be gathered. The determination of the therapeutic efficacy in animal models larger than those currently used (rodents) is also critical. Future prospects point out the increasing use of phototherapy and immunotherapy (in substitution of chemotherapy) and combination therapies as a new direction in cancer treatment. Injectable poloxamer hydrogels will gain prominence not only for drug delivery but also in fields such as tissue engineering and cartilage repair.

Finally, the concluding article offers a comprehensive overview and a critical analysis of several issues related to the use of hydrogels for skin regeneration and wound healing. After enumerating the advantages of hydrogels in wound care, the authors identify the most commonly used polymers in this field and provide a detailed description of their chemical structure, origin and extraction methods, as well as of their intrinsic properties and role in the wound healing process. Whenever possible, the authors provide explanations on the mechanisms of action of individual polymers and their effectiveness; the advantages and limitations of the different polymers are also discussed. Strategies for promoting wound healing, exploiting the intrinsic potential of hydrogels, are presented. These include the use of polymers that stimulate angiogenesis, or the incorporation of bioactive agents, such as drugs, antimicrobial substances or growth factors, which provide additional functionalities to the hydrogels. The usefulness of hydrogels as a 3D matrix for cell culture is also discussed, with particular focus on their potential to support the loading and recruitment of cells to the wound site, where they can proliferate and give rise to new tissue. Finally, future steps in the advancement of hydrogels for wound healing are critically analyzed with particular emphasis on the need for the material to be effective, safe, and environmentally friendly. 

In conclusion, while substantial progress has been made in hydrogel development, overcoming the remaining challenges requires a continued interdisciplinary collaboration between specialists in different areas. The emergence of cutting-edge technologies will help to address issues related with biocompatibility, mechanical properties, enhanced functionality, and production efficiency, leading to the next generation of hydrogels with even greater potential to revolutionize healthcare. Ongoing research is pushing the boundaries of what hydrogels can achieve, exploring new directions [6] (Figure 3) such as **self-healing hydrogels** that repair themselves after damage [7], **bioactive hydrogels** that promote specific cellular responses [8] and **smart hydrogels**, which respond to environmental stimuli, such as pH, temperature, or light [9]. The latter have raised particular interest, due to their potential of enabling adaptive tissue engineering scaffolds and/or controlled and targeted release of drugs and other active agents, enhancing therapeutic outcomes. Current research trends also include novel **nanoarchitectured hydrogels**, incorporating nanoparticulates (as a second phase within the system) capable of accurately controlling drug delivery [10]. Recently, the potential of **three-dimensional printing of hydrogels** in producing living tissue structures or organs [11], or customized dosage forms [12], has been noted. 

Hydrogels will play a pivotal role in achieving more sophisticated medical treatments and devices, opening possibilities for personalized medicine, improved patient outcomes, and innovative solutions to complex medical challenges.

## Figures and Tables

**Figure 1:**
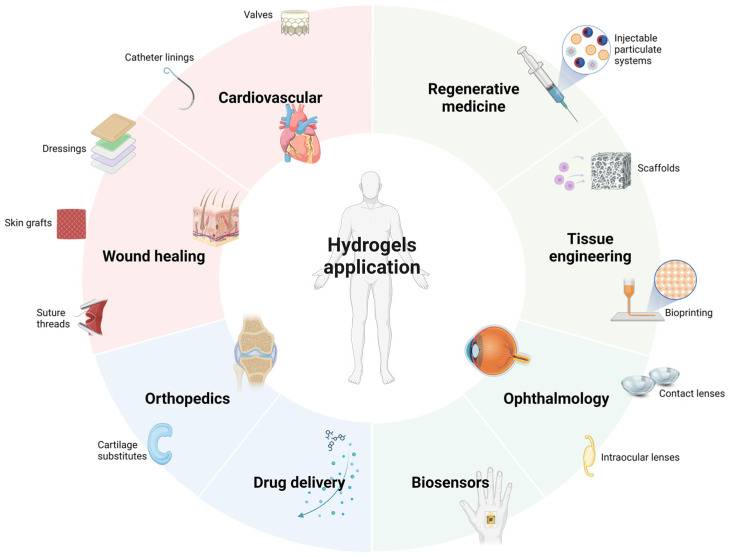
Main areas of application of hydrogels at the biomedical and pharmaceutical level.

**Figure 2:**
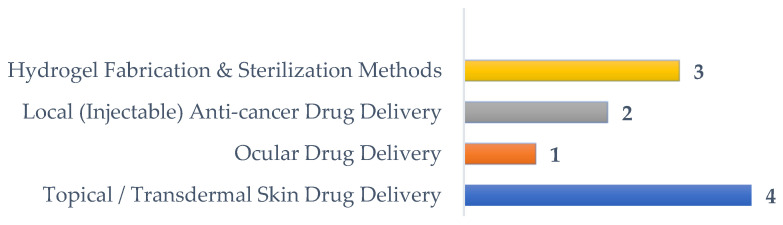
Topics covered in this Special Issue.

**Figure 3:**
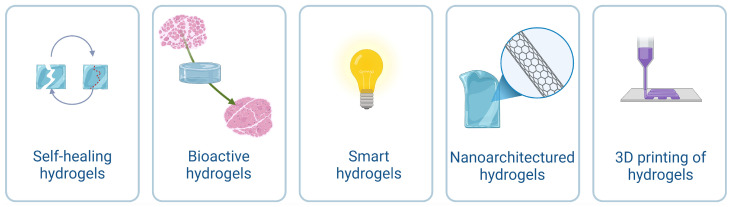
Trends in hydrogel research for biomedical applications.
